# Patriotism and Personal Service

**Published:** 1915-12-18

**Authors:** 


### Patriotism and Personal Service.

### How the Giving Public were Recruited.

The voluntary hospital system has always based its claim to support as much on the value to the individual worker of the self-sacrifice and personal service which it demands of him, as on the treatment and charity which are the benefits bestowed by it upon the individual sufferer. If it were necessary to prove by reference to the great facts of history the eternal basis on which this gospel of personal service rests, it would be necessary merely to consider the one outstanding lesson which the war has taught. That lesson in its broadest aspect is the need for personal service in every individual of the nation. But the effect of this lesson is even wider still. The real thing which the war has taught us is that this gospel of service hardly needs preaching now, for there is evidence of the awakening of a whole people, and, in consequence, a union of hearts and a common determination.

In other words, there is beginning to be observed a moral regeneration—the kind of idealism which has always made it a point of honour to the best type of officer, from Cæsar downwards, to demand no sacrifice of his men which he is not the first to share with them. And if we can only say that this result is beginning to be seen, that is because the effect of the war has necessarily come home to the English people more slowly than to any other belligerent. Great Britain is the reserve of the Allied cause. That is to say, the supreme stress which Belgium, France, and soon afterwards Russia, had to face, is only being felt in England in the later stages of the war. With every month that passes, the nation is beginning increasingly to feel the strain, and we can set no limit at present to the demands which we shall have to meet.

But the regeneration has begun for all that. Any village church will show how men of every class and rank have left to serve the nation, and the list of honoured names which hangs in the porch, and the red crosses which mark the cottage windows, all show how one heart and one mind are animating each of these communities. It is, of course, the same in the towns. It is the same, too, significantly enough, among the women of the families of the upper middle class—the most conservative centre in our national life. These women in the past have mainly lived and died untouched by the strong currents of life around them. They have read of events which they have not shared. They have discussed changes which they have not helped to bring about. In the midst of the flux and eddy without these women have, as a rule pursued their lives at home unaltered, even unaffected, by the current. Now all is changed. The home itself has often been enlarged and revivified. In a hundred and one beneficent ways, of which nursing has been the most popular but not by any means the only example, the women who have had no profession, except that open to them through married life and the care of a family, have now, especially the unmarried, entered on the fuller, ampler existence of professional occupation. Serving at buffets, looking after refugees, munition-making, cooking in V.A.D. hospitals; these and many other occupations are now filled with women, eager, busy, alive, and at work. This phenomenon is the most significant indication of the change of heart and solidarity of purpose which the war has brought to birth. The lives of women, through circumstances, have been in the past largely impervious to national currents. Now that their lives have been swept into the national stream, we can truthfully point to a united and electrified nation. "The power which maketh men to be of one mind in an house," to use the fine old phrase, has opened the doors of the most closely guarded homes, and men and women, in equal streams at last, have emerged bent on the fulfilment of the national purpose. The gospel of personal service has been the inspiration of each, and yet few of the new converts to it realise that the new joy which has come into their lives is only what the voluntary system of hospital support has been preaching year in year out, in time of peace.

Yet the great reward which the hospitals now have, the magnificent tribute which they are paying to the nation, is as much in the long inculcation of giving, which they have developed into a national habit, as in their actual service to the sick and wounded. The wonderful sums achieved by the Red Cross funds are no mere expression of a national impulse. They are, truly regarded, the flood-tide of a stream which has sprung from the appeal of the hospitals, whereby a large proportion of the nation has become accustomed to devote a regular part of its income to the cause of hospitals. By this we mean that the voluntary system has so permeated the national life that when any rich or poor man felt the desire to devote some of his savings to public purposes, hospitals had long become the first object which naturally presented itself to his mind. To have achieved that position in time of peace made the financial needs of Red Cross work virtually secure in time of war.

This financial position of affairs we mention here because it is but another result of that spirit of regeneration and national solidarity which we see to-day as one of the blessings which the war has brought with it. At Christmas time, which is the Festival of the Family, it is well to insist on this blessing, for the nation has become a family, every citizen experiencing that sense of kinship, good will, forbearance, and sacrifice which members of a family should feel towards each other. This, in the larger sense, is what patriotism means. Personal service, the desire to serve, is at its root, and the chief glory of our hospital system to-day is that the whole nation is learning to practise the gospel which the hospitals have preached. The family spirit of goodwill, the Christmas message, is now inspiring the whole nation in its common task.

